# 2439 Depression, anxiety, and planning for the future: Associations with advance care planning

**DOI:** 10.1017/cts.2018.160

**Published:** 2018-11-21

**Authors:** Ryan McMahan, Evan Walker, Rebecca Sudore

**Affiliations:** 1 San Francisco School of Medicine, University of California, CA, USA; 2 Department of Internal Medicine, University of California, San Francisco, CA, USA; 3 Department of Medicine, San Francisco VA Medical Center, Division of Geriatrics, University of California, San Francisco, CA, USA

## Abstract

OBJECTIVES/SPECIFIC AIMS: Millions of diverse, older adults live with serious and chronic illness for which they will face complex, ongoing medical decisions. Advance care planning (ACP) has been conceptualized as a health behavior that supports adults in understanding and sharing their values, goals, and preferences for future medical care. Depression and anxiety are known barriers to participation in health behaviors. It is unknown whether depression and anxiety are associated with ACP participation or with patients’ values for future medical care. Understanding whether depression and anxiety are associated with ACP would be important to tailor ACP interventions. METHODS/STUDY POPULATION: In total, 908 English-speaking and Spanish-speaking participants ≥55 years of age were recruited from a San Francisco county hospital. We measured depression (Patient Health Questionnaire 8-item scale) and anxiety (Generalized Anxiety Disorder 7-item scale), dichotomized into none-to-mild Versus moderate-to-severe. We measured ACP engagement using a validated survey of Behavior Change Processes (e.g., knowledge, self-efficacy, readiness; 5-point Likert) and Action Measures (e.g., ask, discuss, and document one’s wishes; yes/no). We elicited values concerning life extension categorized as “life is always worth living no matter the health situation” Versus “some health situations would make life not worth living.” To explore associations, we usedχ^2^, Mann-Whitney tests, linear and logistic regressions. RESULTS/ANTICIPATED RESULTS: Mean participant age was 64 years±6, 80% were non-White, 40% had limited literacy, 45% were Spanish-speaking, and the prevalence of depression and anxiety was 12% and 10%, respectively. Depression and anxiety were not associated with ACP Engagement, *p*>0.05. However, participants with depression had an increased odds of reporting “some health situations would make life not worth living” than those not depressed, *p*=0.02. In multivariate linear and logistic regression, controlling for age, gender, literacy, and health status, having depression increased the odds of not valuing life extension OR 2.9 (CI: 1.7–4.9). Anxiety was not associated with values concerning life extension, *p*>0.05. DISCUSSION/SIGNIFICANCE OF IMPACT: Depression and anxiety were not associated with prior ACP engagement suggesting engaging patients in ACP does not increase these conditions. However, depression was associated with an increased odds of not valuing life extension and, therefore, may influence treatment choices. Longitudinal randomized controlled trials of an ACP intervention are currently underway to investigate these associations further.

